# An Improved Microscope

**Published:** 1851-06

**Authors:** 


### AN IMPROVED MICROSCOPE.

The difficulties inherent in the most approved microscopes of English and French manufacture have been frequently felt by all who have used those instruments, whether manufactured by Ross, Pritchard, Oberhauser, Nachel, or Chevalier. During the past winter, Professor Silliman informed us of an American invention which promised the removal of the difficulties to which we refer. We have recently enjoyed a pleasant and instructive intercourse with the distinguished inventor of this improved microscope—Dr. J. Lawrence Smith, and we have found the instrument all that can be desired. For the prosecution of microscopic investigations of any kind, but especially of a chemical or geological character, Dr. Smith's microscope excels any one we have seen, and must command the confidence of all who pay any attention to microscopic studies.

In the absence of drawings it is difficult to describe an instrument in such a way as to make its character properly understood. But we feel so much the importance of microscopic researches, as aids to pathology and as indicæ to therapeutics, and are impressed so fully with the value of Dr. Smith's invention, that we shall endeavor to describe the advantages of that invention.

The peculiarities of Dr. Smith's microscope consist in having the object placed above the objective glass, instead of beneath it, and the ray of light passing into the objective is deflected 150° before it reaches the eye. The deflection is accomplished with the aid of a single prism with two reflecting surfaces. The occular extremity and the stage of the microscope are so placed that they both come beneath the eye, and can be regarded almost at one and the same time.

The advantages offered by this arrangement are best seen where manipulations are required, either of a chemical or electrical character.

The stage of the microscope is perfectly unembarrassed, and is so situated with reference to the eye, that the operator is enabled to work with the greatest facility, without interfering in the least with his observations in the instrument.

Even for general purposes this microscope is superior to the microscopes commonly in use, and this could be readily understood on seeing the instrument, or a drawing of it. The most persistent use of Dr. Smith's instrument gives no fatigue to the observer. We may examine an object microscopically, and watch the effect of heat, or of electricity upon it, all at the same time, and vapor, so detrimental to microscopic observation in other instruments, does no harm in this.

Combined with this microscope is a method for measuring the dimensions of objects and the angles of crystals, that is more simple and accurate than any yet known, and this method can be adapted to the instruments in common use. Nachel of Paris has already applied this method to several instruments of his mounting. Our friend Dr. Clapp, of New Albany, the distinguished cultivator of natural science, examined Dr. Smith's invention a few days since, and speaks in the warmest terms of admiration of its excellence. The method of measuring the dimensions of objects, and the angles of crystals, impressed Dr. Clapp very favorably. A detailed description of Dr. Smith's instrument, with drawings, will be published shortly.

We have enjoyed many opportunities of using Dr. S's instrument, in the course of a series of chemical analyses, of urinary calculi, conducted by Dr. Smith, and we speak understandingly, when we say that it is far superior to those known as Oberhauser's or Nachel's. Dr. Smith is one of the most thoroughly qualified gentlemen in chemical knowledge that we know, and the readers of this Journal will be pleased to learn, that he has promised us a paper on Urinary Calculi. It cannot fail to be an interesting one. B.

